# Nanomechanics and Plasticity

**DOI:** 10.3390/nano12213807

**Published:** 2022-10-28

**Authors:** Haifei Zhan

**Affiliations:** 1 College of Civil Engineering and Architecture, Zhejiang University, Hangzhou 310058, China; zhan_haifei@zju.edu.cn; 2 School of Mechanical, Medical and Process Engineering, Queensland University of Technology (QUT), Brisbane, QLD 4001, Australia

Nanomaterials and nanostructures are continuously driving technology revolutions in broad engineering fields, such as defense [1], aerospace, electronics [2], biomedical [3,4], energy [5], and other high-end sectors. Landmark nanostructures, such as fullerene [6], carbon nanotube (CNT) [7], and graphene [8], have significantly promoted the growth of nanomaterials and triggered novel applications. For instance, there are increasing interests in the synthetic nanochannels (e.g., graphene nanochannels), which possess unique transmission behavior and show appealing prospects for nanofiltration [9,10], water desalination [11,12,13,14], and energy storage [15,16]. Through atomistic simulations, Li et al. [17] investigated the permeability of a hexagonal diamond nanochannel for a NaCl solution. To facilitate various engineering implementations, a comprehensive understanding of their mechanical properties and deformation mechanisms is usually a prerequisite, which can be acquired through nanoscale experiments and atomistic calculations.

Extensive works have been conducted to extend the concepts/theories established at macro-scale to nanoscale, in order to describe the mechanical behavior of nanomaterials. For instance, surface effects and axial extension have been incorporated in the beam theory to describe the bending and vibration of nanowires [18,19], which are the building blocks for nanoelectromechanical systems. Considering the coupled loading scenarios, Lu et al. [20] investigated the deformation mechanisms of nanowire under coupled tension-torsion loads. Pan et al. [21] derived the exact solutions for torsion and warping of axial-loaded beam-columns in order to avoid the underestimation of the torsional stiffness of thin-walled nanostructures. With the advancement of fabrication technology, more complex nanostructures have been reported, such as heterostructures, Janus structures, helical/spiral structures [22], nanoscrolls [23], and three-dimensional networks [24]. These complex structures exhibit unique physical and chemical properties. For instance, Yang et al. [25] found that the Janus WSSe and MoSSe monolayers exhibit a strong mechanical anisotropy under tension. 

A common utilization of nanomaterials is reinforcement for engineering materials, which includes polymers, fibers and metals [26]. Extensive works have employed low-dimensional nanomaterials to enhance the mechanical and thermal properties of composite materials [27,28]. For instance, the CNT has excellent mechanical properties, which is promising for artificial muscles and flexible electronics, while the mechanical performance of CNT fibers/bundles depend on various factors, such as the alignments and compositions of constituent CNTs as shown by Wei et al. [29]. The excellent mechanical properties of nanomaterials also make them promising for extreme applications, such as the protective shields that alleviate the damage from the hyper-velocity impact. In this regard, Xia et al. [30] investigated the deformation and penetration mechanisms of titanium carbide MXene nanosheets. Studies reveal that many factors affect the mechanical performances of polymer composites synergistically, such as the type, percentage, alignment, dispersion, and functional groups of the nanofillers [31]. Investigations show that CNT acts as a skeleton in the poly phenylene terephthalamide polymer that enhance both the strength and modulus [32], and the graphene foam can remarkably increase the storage modulus and loss modulus of the polydimethylsiloxane polymer [33]. 

Besides nanomaterials, plenty of works have investigated the atomistic underlying mechanisms for the mechanical behaviors of engineering materials, which provides useful guidelines for the design of high-performance materials. For example, Wu et al. [34] probed the penetration process of aluminum nanorod through atomistic simulations, and Wan et al. [35] assessed how the initial void influence the damage characteristic of single crystal aluminum under shock loading. Interestingly, Jiang et al. [36] found that the presence of copper nanoparticle will trigger the formation of regular stacking fault in the single crystal aluminum under shock compression. Based on the smoothed particle hydrodynamics (SPH) method, Wu et al. [37] discussed the differences in the fragmentation characteristics between the microscopic (atomic scale) and macroscopic scales under hypervelocity impact. In real applications, materials are usually exposed under a complex loading environment, e.g., electronics experience cyclic thermal and mechanical loadings during service [38]. As such, it is crucial to explore the responses of materials under coupled loading. For example, Zhao et al. [39] established a multiscale model to investigate the mechanical–thermal responses of woven composites. There is also increasing attention being devoted to the strain engineering of nanomaterials. For instance, Yang et al. [40] reported that proper strain can modulate the valley polarization in VS2 heterostructures. Other works also reported the strain tailored thermal transport properties [41] and interfacial thermal conduction of nanostructures [42].

In summary, this Special Issue of *Nanomaterials* entitled “Nanomechanics and Plasticity” compiles a series of original research articles that explore the mechanics at nanoscale for different advanced materials. We are confident that this Special Issue will provide the reader with an overall view of some of the latest prospects in the nanotechnology field.

## Data Availability

Not applicable.
